# Design of Extruded Nanostructured Composites via Decoupling of the Cellulose Nanofibril/Poly(butylene adipate-*co*-terephthalate) Interface

**DOI:** 10.1021/acsami.4c17899

**Published:** 2024-12-23

**Authors:** Angelica Avella, Maria Rosella Telaretti Leggieri, Alexandros Efraim Alexakis, Eva Malmström, Giada Lo Re

**Affiliations:** † Department of Industrial and Materials Science, Chalmers University of Technology, SE-412 58 Gothenburg, Sweden; ‡ Wallenberg Wood Science Centre, Chalmers University of Technology, Kemigården 4, SE-412 96 Gothenburg, Sweden; § Division of Coating Technology, Department of Fibre and Polymer Technology, School of Engineering Science in Chemistry, Biotechnology and Health, KTH Royal Institute of Technology, Teknikringen 56-58, SE-100 44 Stockholm, Sweden; ∥ Wallenberg Wood Science Centre, Department of Fibre and Polymer Technology, KTH Royal Institute of Technology, Teknikringen 56-58, SE-100 44 Stockholm, Sweden

**Keywords:** diblock copolymers, cellulose nanofibrils, nanocomposites, poly(butylene adipate-*co*-terephthalate), interface design

## Abstract

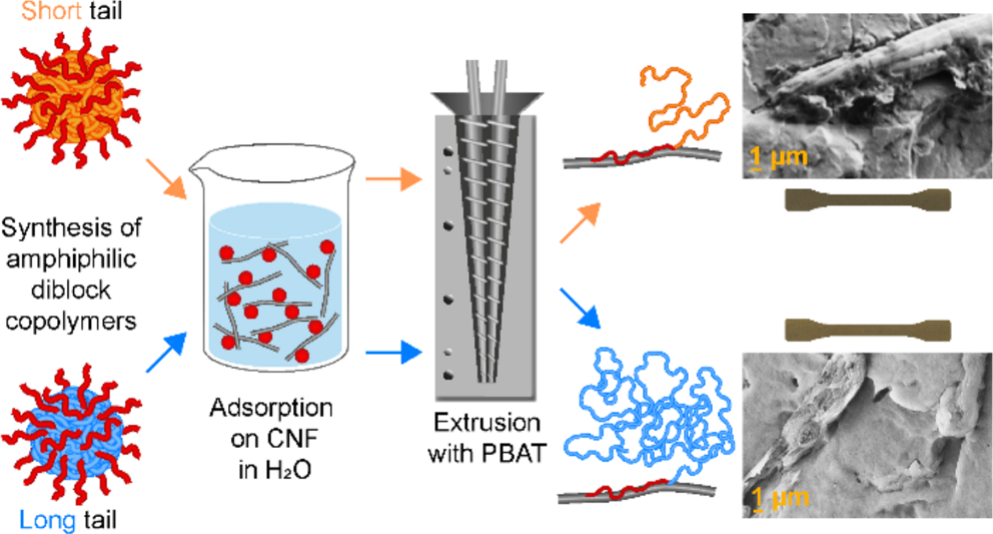

The full exploitation of the outstanding mechanical properties of cellulose nanofibrils (CNFs) as potential reinforcements in nanocomposite materials is limited by the poor interactions at the CNF–polymer matrix interface. Within this work, tailor-made copolymers were designed to mediate the interface between CNFs and biodegradable poly(butylene adipate-*co*-terephthalate) (PBAT), and their effect on extruded nanocomposite performance was tested. For this purpose, two well-defined amphiphilic anchor–tail diblock copolymer structures were compared, with a fixed anchor block length and a large difference in the hydrophobic tail block length. The aim was to evaluate the impact of the copolymers’ chain length on the nanocomposite interface. The presence of amphiphilic diblock copolymers significantly improved the mechanical properties compared to those of PBAT nanocomposites containing unmodified CNFs. In particular, the copolymer with a longer tail was more effective for CNF–PBAT dispersion interactions, leading to a 65% increase of Young’s modulus of neat PBAT, while retaining high deformability (670%). The results provide insights into the effectiveness of a waterborne third component at the CNF–matrix interface and its structure–property relationship.

## Introduction

A homogeneous dispersion of nanoparticles in the polymer matrix is the key to achieve reinforcement in nanocomposite materials at the nanoscale, together with a strong nanoparticle–matrix adhesion, which ensures stress transfer across their interface. Understanding essential parameters for tuning the performance at the interface between a thermoplastic polymer and reinforcing nanocellulose is the focus of this study.

Insufficient control over the nanocellulose–matrix interface at the nanoscale is often what limits the reinforcement potential of cellulose nanomaterials such as cellulose nanofibrils (CNFs) in polymer nanocomposites. Failure to achieve high-performance nanocomposites is a result of nanofibril self-aggregation and consequent pull-out phenomena due to poor interactions at the interface. CNFs, biobased and biodegradable fibrillar nanoparticles with unique properties (aspect ratio ≈ 100–300, elastic modulus ≈ 100 GPa, strength ≈ 4 GPa, combined with low density ≈ 1.7 g/cm^3^)^1−3^ are as attractive as they are challenging when selected as reinforcing nanoadditives. Their colloidal stability is commonly achieved by surface modification with negatively charged groups (e.g., carboxylate) and is limited to water dispersions, which hampers their use in conventional thermoplastic polymers, which are relatively hydrophobic. The interparticle interaction, driven by hydroxyl groups on the CNF surface, is a double-edged sword: it is advantageous, allowing for the formation of a load-bearing percolating network when CNFs are efficiently dispersed, but it leads to a strong tendency for self-aggregation when CNFs are processed in hydrophobic media in the absence of a suitable strategy for controlling the hydrophilic–hydrophobic interface. Compared to other cellulose products, CNFs are the most challenging to disperse due to their large aspect ratio, therefore they represent a model system to test interface design.^4^

The existing strategies for dispersing nanocelluloses in hydrophobic polymer matrices range from their covalent functionalization with hydrophobic moieties to the adsorption of surfactants or functional polymers.^5−7^ A promising strategy for mediating the hydrophilic–hydrophobic interface between nanocellulose and thermoplastic polymers relies on surface engineering by introducing an amphiphilic third component. This route has been explored in a few studies.^8−10^ The materials commonly used for this purpose are amphiphilic block copolymers functioning as anchor–tail systems. In these copolymer structures, the hydrophilic block effectively adsorbs on the nanocellulose surface, serving as the anchor, while the hydrophobic block mixes and ideally entangles with the matrix, serving as the dispersing tail.

As the hydrophilic anchor block, both noncharged polymers such as poly(2-hydroxyethyl methacrylate)^11,12^ and cationic polyelectrolytes such as poly(2-(dimethylamino)ethyl methacrylate) (PDMAEMA)^8,9^ have been used. Cationic polyelectrolytes have the advantage of ensuring a strong adhesion onto negatively charged nanocelluloses based on electrostatic adsorption, which leads to favorable conditions for stress transfer. At the same time, a dynamic ionic bonding may be established at the interface, leading to a more efficient reinforcement.^9,13,14^ Amphiphilic block copolymers have the advantage of being dispersible in water, where they typically self-assemble into micellar nanoparticles.^15−17^ This allows for their adsorption onto CNFs directly in water dispersion through a nontoxic and scalable process.

The quest for an optimal dispersion of CNFs in hydrophobic polymer melts is at the core of a large number of studies. An important field of application for CNFs is related to their incorporation in biodegradable polymers to improve or tune their mechanical properties, expanding their potential range of uses. Herein, the aim is to deepen the understanding of the hydrophilic–hydrophobic interface between CNFs and a thermoplastic biodegradable polymer when an amphiphilic third component is used to mediate their interface. These insights are valuable to understand how to optimally exploit biobased nanostructures and their interactions with the matrix for improving the performance of biodegradable nanocomposites.

Two model diblock copolymers were designed with a fixed anchor block length and a substantial difference in the length of the hydrophobic tail block. The aim is to study the effect of the tail length on the CNF–matrix interaction by replacing their interface and introducing two new interfaces: copolymer–CNF and copolymer–matrix. In our previous study,^14^ a statistical copolymer was designed to adhere to the CNF surface to form a core–shell structure, and the adhesion with the polymer matrix was achieved by reactive extrusion. In this work, we synthesize diblock copolymers where the interaction with negatively charged CNFs is secured by dynamic ionic bonding with the quaternized block, while the interaction with the polymer matrix is promoted by the relatively more hydrophobic block. Moreover, the chain entanglement contribution to the copolymer–matrix interaction is evaluated by tuning the tail length of the hydrophobic block above or below the entanglement point with poly(butylene adipate-*co*-terephthalate) (PBAT).

Amphiphilic micellar diblock copolymers were advantageously adsorbed onto CNFs in water dispersion, aiming for a homogeneous distribution of the nanoparticles throughout the surfaces of CNFs upon subsequent water removal. Extrusion of the nanocomposites was carried out with wet feeding, to ensure optimal conditions for the dispersion of CNFs in the matrix.^18^

Poly(butylene adipate-*co*-terephthalate) (PBAT) was selected as the polymer matrix since it is a growing biodegradable alternative to conventional thermoplastic polymers^19^ and has limited hydrolysis when processed with water. PBAT is highly deformable, hence interesting for packaging applications, but its potential uses are limited due to low Young’s modulus and tensile strength with respect to other polymers of common use, such as high-density polyethylene.^20^ Few studies have explored the use of nanocellulose in PBAT nanocomposites with the aim of improving the matrix properties.^20−23^ Hou et al.^21^ and Lai et al.^20^ carried out surface modifications of CNFs in organic solvents, introducing epoxide or amine functional groups, followed by extrusion in the dry state. Both works produced nanocomposites with less than 2 wt % CNFs. Edlund et al.^23^ developed a method for coating CNFs with fatty acrylate polymers through admicellar polymerization and extruded nanocomposites with 5 and 20 wt % CNFs.

The results of this work deepen our current knowledge of how to design an effective third component for mediating the interface between a hydrophobic polymer matrix and hydrophilic nanofibrils. This study contributes to the exploitation of natural nanoreinforcements in polymer nanocomposites, widening their application and therefore motivating their commercial production.

## Results and Discussion

The aim of this study is the interface design of PBAT/CNF nanocomposites through mediation with amphiphilic diblock copolymers. Two copolymers were synthesized with different hydrophobic tail lengths to study their interactions with the polymer matrix. The copolymers were dispersed in water, where they self-assembled into nanosized micelles. Afterward, the adsorption onto the negatively charged CNFs was driven by ionic interactions with the cationic blocks of the copolymers. Modified or neat CNFs were blended with PBAT via water-assisted extrusion, and mechanical and morphological analyses were carried out to investigate the influence of the interface design on the nanocomposite properties.

### Copolymer Synthesis and Characterization

**Figure 1:**
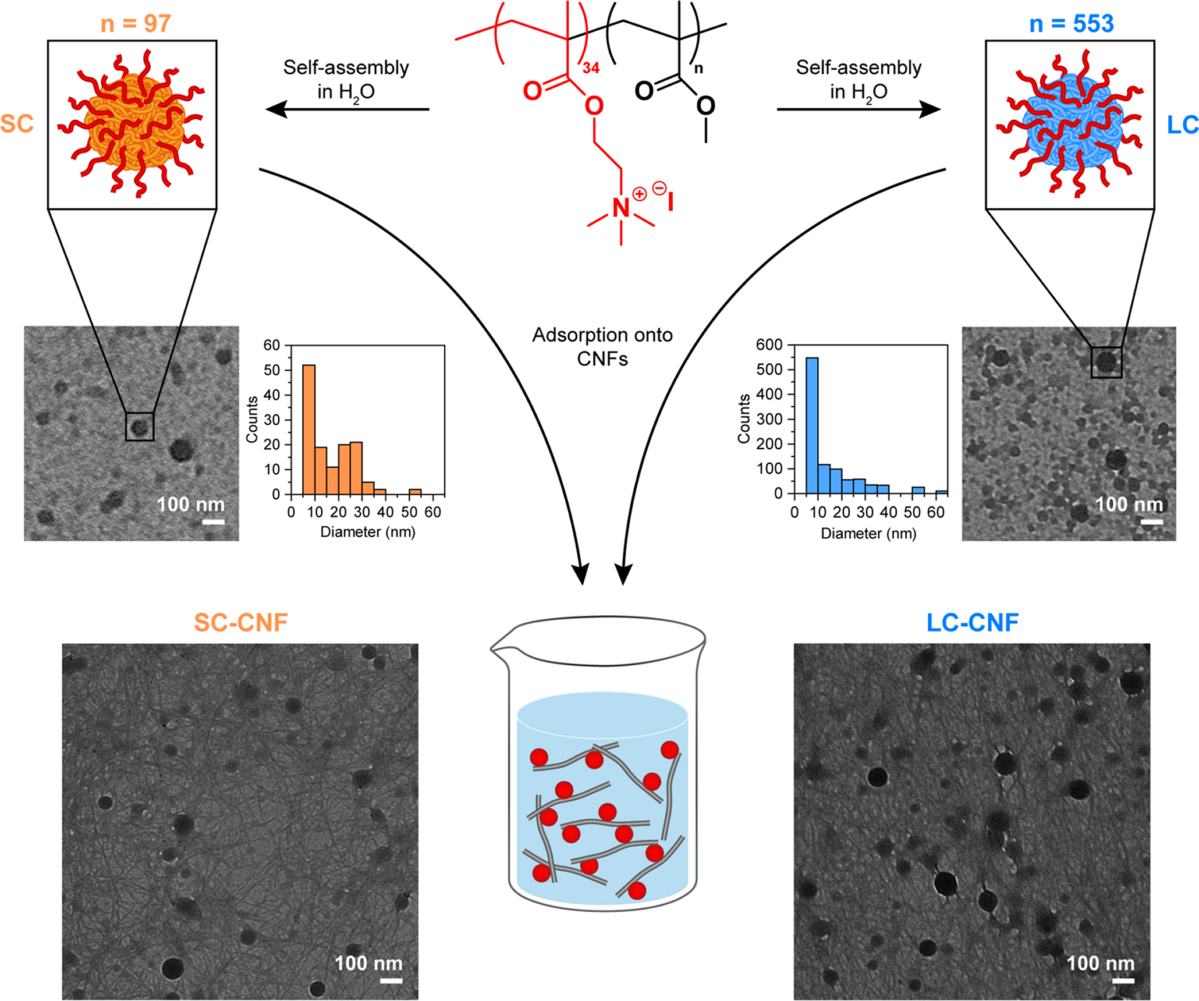
Schematic overview of the copolymers’ structure, self-assembly into spherical nanoparticles in water, and adsorption onto CNFs in water dispersion. The dimensions of the components in the sketches are not in scale. Transmission electron microscopy (TEM) micrographs of SC and LC nanoparticles are reported, together with their size distribution estimated based on scanning electron microscopy (SEM) images (Figure S5), as well as TEM micrographs of SC and LC adsorbed onto CNFs.

Two diblock copolymers with a cationic polyelectrolyte block were designed using the same PDMAEMA hydrophilic anchor block and two different lengths of the poly(methyl methacrylate) (PMMA) hydrophobic block: SC, *short copolymer*, and LC, *long copolymer* (Figure 1 and Table 1). Size-exclusion chromatography (SEC) is not suitable for absolute assessment of the molecular weight of PDMAEMA-*b*-PMMA diblock copolymers (Figure S1 and Table 1) due to the difference in hydrodynamic volume compared to the standards available. The average degree of polymerization (DP) of the hydrophobic block and thus the molecular weight of the copolymers (*M̅*_*n*,EA_) were assessed through elemental analysis (EA) (Table 1).

**Table 1:** Physicochemical Properties of the Copolymers Used in This Study

acronym	DP of blocks^a^	*M̅*_*n*,EA_^b^ (g/mol)	*M̅*_*n*,SEC_^c^ (g/mol)	*Đ*^c^	*T*_g_^d^ (°C)	charge^e^ (μmol/g)	diameter^f^ (nm)
SC	*q*PDMAEMA_34_-*b*-PMMA_97_	15,200	35,700	1.08	123	855 ± 6	19 ± 10
LC	*q*PDMAEMA_34_-*b*-PMMA_553_	61,900	70,000	1.15	127	171 ± 1	18 ± 12

a The degree of polymerization (DP) of the initiating block was assessed by proton nuclear magnetic resonance spectroscopy (^1^H NMR), while the DP of the extension block by EA.

b Determined before quaternization by calculations based on EA.

c Determined before quaternization by SEC in DMF.

d Determined by DSC.

e Determined by PET.

f Determined by image analysis of scanning electron micrographs processed using ImageJ software.

The glass transition temperatures (*T*_g_) of the copolymers, detected by the second heating run in differential scanning calorimetry (DSC), are consistent with the *T*_g_ of the PMMA block alone (Figure S2 and Table 1).^24^

The copolymers, self-assembled into spherical nanoparticles in water, were found to have significantly different surface charge densities by polyelectrolyte titration (PET) (Table 1). The different ratio between the charged anchor block (with a constant length in both copolymers) and the hydrophobic tail is responsible for higher charge per gram measured for the short copolymer.

**Figure 2:**
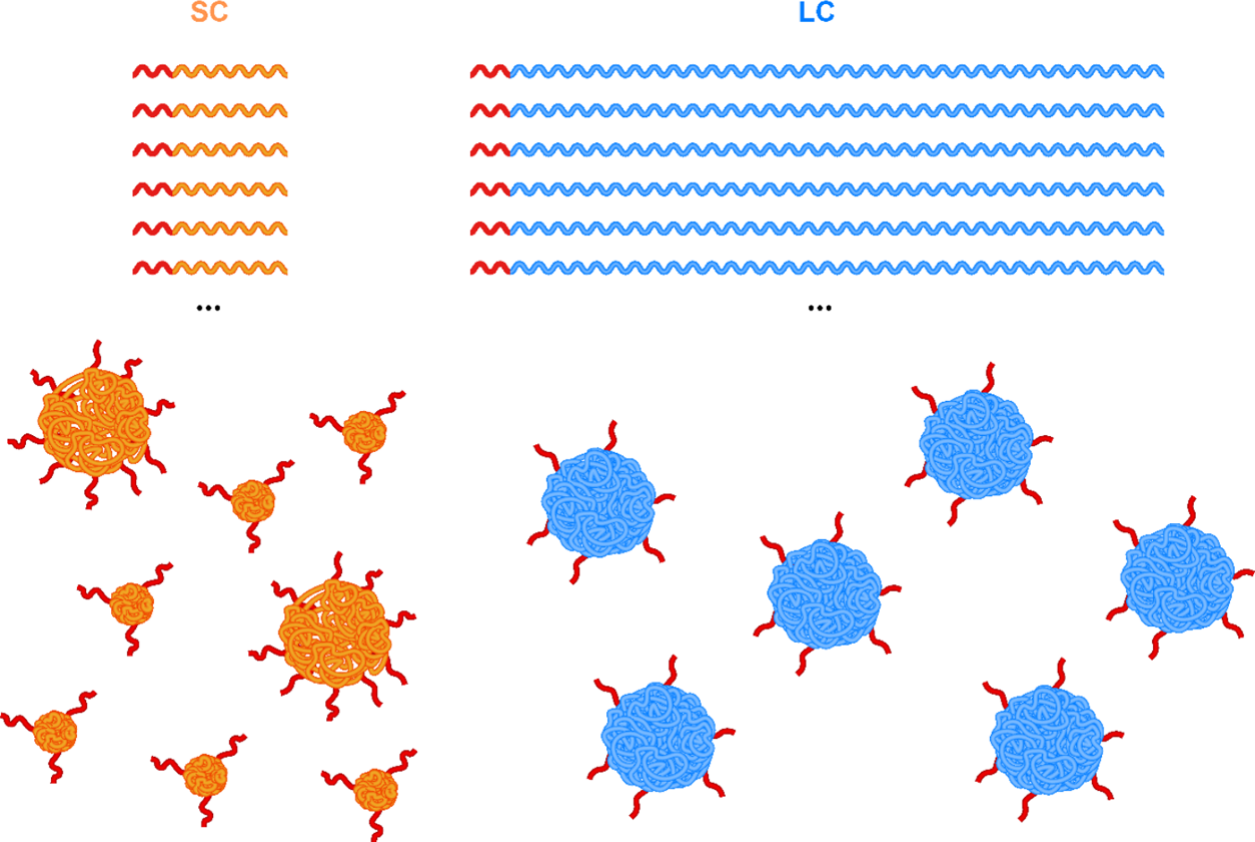
Scheme of the hypothesized self-assembly of the short (SC) and long (LC) copolymers in water. The number of represented copolymer chains (upper panel) exemplifies the 1:1 charge ratio. The proposed mono- and bimodal size distribution (bottom panel), for LC and SC respectively, is a simplification of a possible broader size distribution of waterborne sub- to nanoparticles.

The nanoparticles were imaged by transmission electron microscopy (TEM) (Figures 1 and S3) and scanning electron microscopy (SEM) (Figure S4). The image analysis of the SEM micrographs indicated that the nanoparticle count for the long copolymer is 1 order of magnitude higher than the count of the short one (histograms in Figure 1), and an average nanoparticle diameter in the dry state was the same for both copolymers (≈18 nm) (Table 1 and Figure S5). DLS in deionized water also indicated similar hydrodynamic diameters of the two copolymers (Table S1). This result has also been observed by Utsel et al.,^25^ who showed that varying the length of the poly(ε-caprolactone) (PCL) in PDMAEMA-*block*-PCL copolymers did not alter the hydrodynamic radius of the copolymer micelles in water. The same work demonstrated that the increase of the hydrophobic block length increased the water contact angle, i.e., increased hydrophobic character. However, it is worth noting that these techniques cannot resolve particle sizes below the nanometer scale, therefore providing a partial morphological description of the systems. The counterintuitive evidence of similar size and the partial count drive the hypothesis of the presence of subnanometric particles that cannot be detected even in the TEM microscopy (Figure 2). This hypothesis explains the few detected particles in the short copolymer, especially considering that the observed dispersions have the same copolymer concentration.

SC and LC, self-assembled into cationic spherical nanoparticles in water, were adsorbed onto CNFs (Figure 1) yielding dispersions of SC-modified CNFs (SC–CNF) and LC-modified CNFs (LC–CNF). The TEM images of SC–CNF and LC–CNF show that the nanoparticles are homogeneously distributed throughout the network of nanofibrils formed upon drying (Figure 1). The complete softening of the copolymers at the processing conditions, required to favor their interaction with the polymer matrix in the melt state, was verified by SEM of the CNFs/copolymer mixtures before and after annealing at 160 °C for 20 min (Figure S6).^9^

To verify that the nanoadditives do not degrade under the conditions selected for processing the nanocomposites, thermogravimetric analysis (TGA) was conducted on CNFs and copolymers. The analysis showed that the onset temperature of thermal degradation (*T*_5%_) of CNFs, SC and LC was above 220 °C in all cases (Figure S7 and Table S2), sufficiently higher than the selected temperature for extrusion (160 °C). This hypothesis has been further confirmed by an isothermal gravimetric analysis at 160 °C for the extrusion time, which did not register any mass loss of the CNFs (Figure S8).

### Nanocomposites Extrusion and Characterization

Wet feeding was chosen as the optimal route to minimize agglomeration of CNFs during extrusion.^18^ The extrusion of all materials was carried out by starting with an amount of water equivalent to 50 wt % of the solid fraction, which was evaporated during processing at 160 °C for 10 min. For reference, neat PBAT was also processed with water, and its molecular weight and polydispersity were measured to assess possible hydrolysis during processing.

SEC analysis shows a slight reduction in the molecular weight of PBAT (*M̅_n_* from 37 to 31 kDa) and an increase in polydispersity (*Đ* from 2.1 to 2.3) when processed with 50 wt % of water, compared to dry processing (Table S3 and Figure S9). These results indicate slight hydrolysis during extrusion, however, the established benefits of wet feeding of cellulose-based nanocomposites are considered to prevail over the hydrolytic effect of water on the matrix.^18^

**Figure 3:**
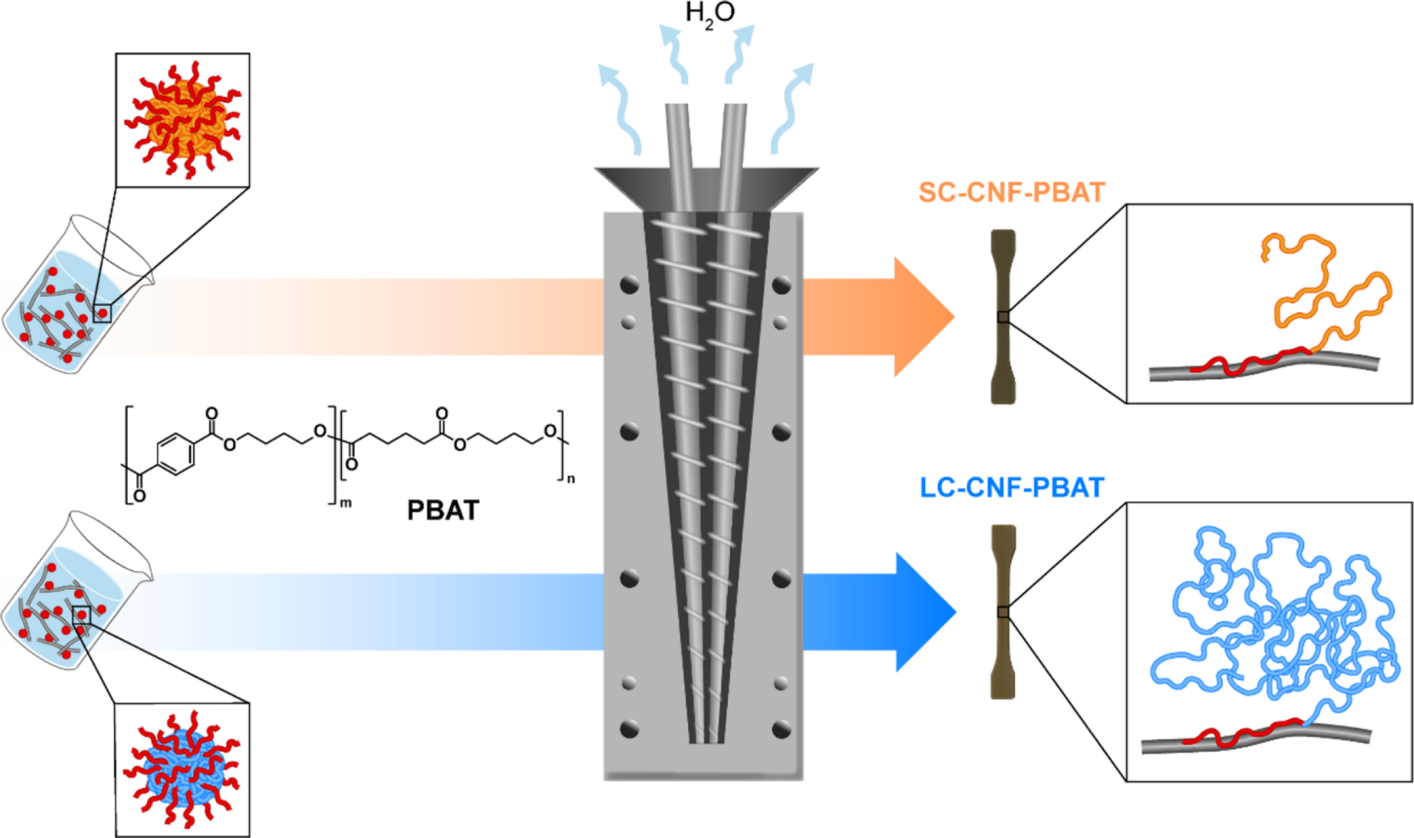
Schematic overview of the extrusion process. In the inset to the right, the copolymers are visually represented as coils with block lengths to scale, while the CNF dimensions are not to scale.

First, a nanocomposite containing 3 wt % CNFs and 3 phr long copolymer was extruded (3LC-3CNF-PBAT entry in Table 2), however, its tensile properties (Table S5) were not significantly different from neat PBAT. To capture a more pronounced effect, the CNF content was doubled in order to tackle the challenges of nanocellulose individualization.^26,27^ The 6 wt % CNF ensured evidence of reinforcement in the nanocomposites, so it was selected as a constant parameter to highlight the mere copolymer effect at the interface. Therefore, nanocomposites with 6 wt % CNFs were extruded with 1.2 wt % SC or 6 wt % LC (Table 2 and Figure 3). The contents were chosen aiming to keep the CNF/copolymer charge density ratio constant, since surface charge is a crucial parameter in the adsorption of cationic nanoparticles onto charged cellulose surfaces.^17^ A nanocomposite with 6 wt % SC was also extruded to have an LC/SC comparison by weight ratio. Reference materials of PBAT extruded with LC, SC, and CNFs were produced to evaluate the effect of the single components.

**Table 2:** List of Extruded Materials Evaluated in This Study

samples	PBAT (wt %)	CNF (wt %)	SC (phr)	LC (phr)
PBAT_dry_^a^	100			
PBAT	100			
CNF-PBAT	94	6		
SC-PBAT	100		1.2	
SC–CNF-PBAT	94	6	1.2	
6SC–CNF-PBAT	94	6	6	
LC-PBAT	100			6
LC–CNF-PBAT	94	6		6
**3LC-3CNF-PBAT**	97	3		3

a Neat PBAT, processed in the absence of water.

After processing, all materials except PBAT turned darker, especially CNF-PBAT (Figure S10). It is well-known that the melt processing of cellulose-based composites leads to discoloration, as explained by oxidation at high temperatures. However, the visual aspects do not always correlate to thermal degradation or loss in mechanical performance.^28^

The thermal properties of the extruded materials were determined by DSC and TGA to understand the effect of CNFs and copolymers on the thermal transitions, degradation, and crystallinity of PBAT (Figure S11 and Table S4). The glass transition and melting temperatures are similar for all materials with no significant differences within the error of the characterization technique. Both CNFs and the copolymers increase the crystallization temperature of PBAT, acting as nucleating agents, while the overall crystalline fraction is not significantly affected. This result indicates that only the crystallization kinetics are modified by the incorporation of CNFs and the copolymers and that changes in the static and dynamic mechanical properties cannot be ascribed to crystallinity variations.

The onset of thermal degradation of the nanocomposites is lower than that of the matrix, reflecting the lower thermal stability of CNFs and copolymers compared to neat PBAT. The main degradation temperature is similar for all materials, indicating that the degradation of PBAT is not affected by the additives.

**Figure 4:**
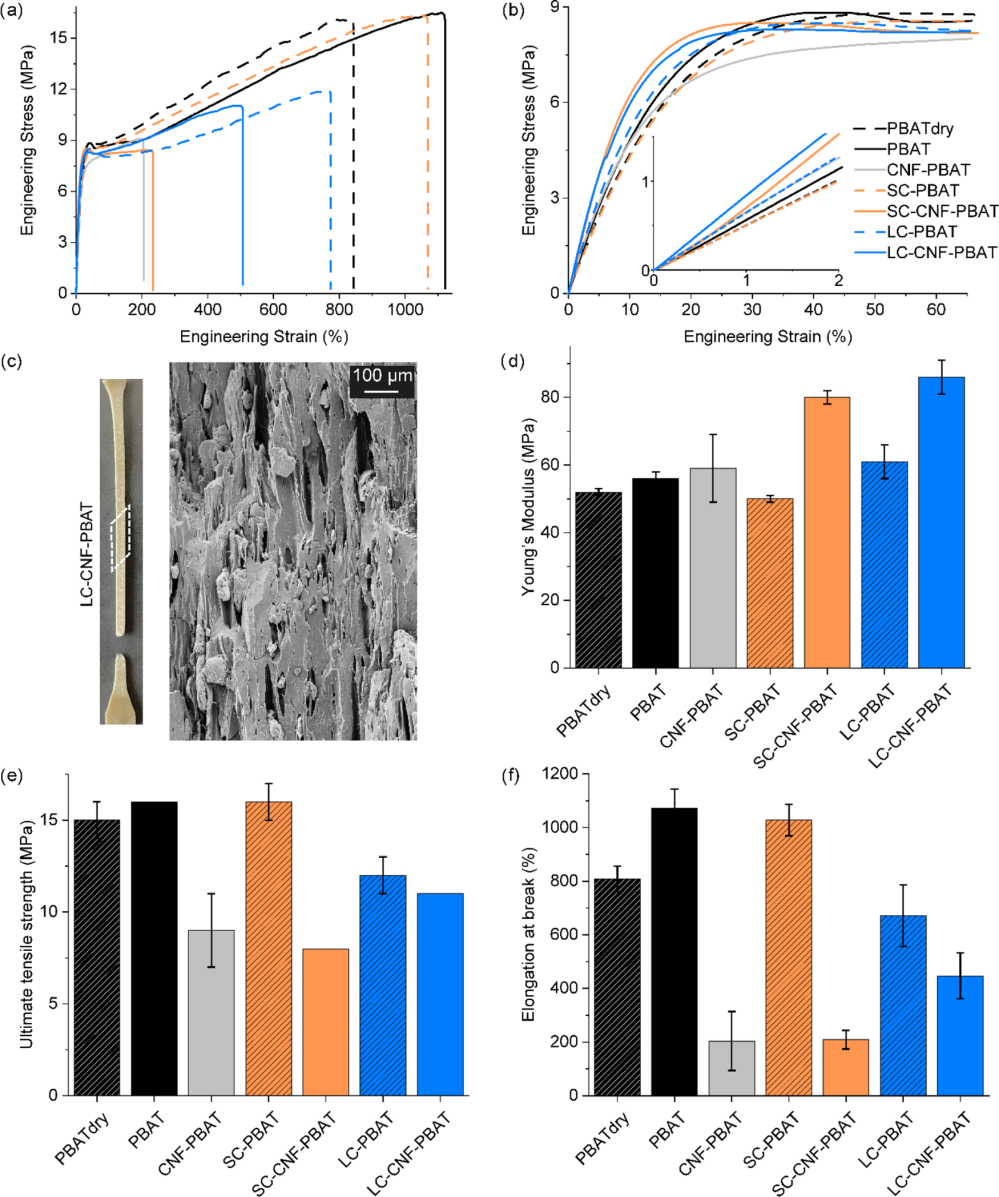
(a) Representative tensile curves of the nanocomposites and their references, with enlargements of (b) the yield point and the linear region. (c) Photograph of an LC–CNF-PBAT specimen after the tensile fracture and scanning electron microscopy of its cryofracture along the tensile direction, showing the presence of crazing. Average values with standard deviation of (d) Young’s modulus, (e) ultimate tensile strength, and (f) elongation at break.

The thermomechanical properties of the nanocomposites were assessed by tensile testing and dynamic mechanical thermal analysis (DMTA) on the injection-molded specimens to investigate how the copolymers influence the CNFs/PBAT interaction (Figure 4 and Table S5).

PBAT is a highly deformable polymer, with elongation at break above 1000% and Young’s modulus ≈ 50 MPa. Extrusion with water increases the deformability of PBAT and slightly improves its stiffness, possibly due to the hydrolysis and recombination occurring during melt processing.

SC alone slightly reduces Young’s modulus of PBAT but does not affect its deformability due to the low amount (1.2 wt %) in SC-PBAT. LC at 6 wt % slightly stiffens and embrittles the matrix due to the higher amount of the copolymer in LC-PBAT than SC-PBAT, suggesting immiscibility between the long copolymer and PBAT.

The addition of CNFs has a slight stiffening effect on PBAT but a significant reduction in deformability. The decrease in elongation at break (ε_b_ of CNF-PBAT > 200%) is however not as dramatic as for other nanocomposites reported in the literature with similar or even lower CNF content.^12,23^ It is worth noting that the mechanical properties of CNF-PBAT have large scattering, an indication of the inhomogeneity of the nanocomposite caused by CNF agglomerates.

The adsorption of the copolymers onto the CNF surface improves the stiffening effect, with 43 and 53% increments in Young’s modulus for SC and LC, respectively, compared to neat PBAT. SC–CNF-PBAT is as brittle as CNF-PBAT, while the elongation at break of LC–CNF-PBAT is more than double. The reported changes are relative to PBAT processed with water to exclude the contribution of water to the mechanical properties of the nanocomposites. For the sake of comparison with the literature, where the reference is dry commercial PBAT, the increment of Young’s modulus in our nanocomposites compared to neat PBAT is 54 and 65% while the reduction of deformability is 74 and 45% for SC–CNF-PBAT and LC–CNF-PBAT, respectively.

The improvement of the mechanical properties can be caused by improved miscibility, i.e., better CNF dispersion and/or better adhesion with PBAT, while the contribution of crystallinity is excluded based on thermal analysis (Table S4). To improve CNF miscibility in PBAT, we designed two copolymers generating two new interfaces, CNF–copolymer and copolymer–PBAT. Both copolymers have identical ionic interaction with the CNFs, due to identical cationic anchor blocks, and different molecular weights of the hydrophobic tail, intended to mediate the interface with PBAT. Both copolymers, when adsorbed onto CNFs, limit self-interaction and self-assembly of CNFs, thus hindering their agglomeration when in the nanocomposites.^29^ The identical hydrophilic anchor justifies the observed increased stiffness of the nanocomposites.^30^ The preserved deformability in the case of LC compared to SC–CNF-PBAT and CNF-PBAT, confirms that a longer hydrophobic tail promotes entanglements with the matrix macromolecules, thus leading to a better copolymer–PBAT interface.

To further investigate this hypothesis and understand whether the poorer efficiency of SC was due to the lower weight compared to LC, a nanocomposite with 6 wt % SC was used as a comparative reference. The adsorption of SC onto CNFs in this ratio (SC/CNF 1:1 by weight) led to a milky gel due to CNF charge saturation (Figures S12 and S13). SEM images of the cryo-fractured surface of 6SC–CNF-PBAT showed an increased number of spherical particles that can be ascribed to the phase separation of free copolymer (not adsorbed onto CNFs) into the nanocomposite (Figure S14). This result is in agreement with the hypothesized self-assembly of the copolymers (Figure 2), as an excessive amount of SC (SC/CNF charge ratio = 5:3) would form a large number of particles that cannot be fully adsorbed onto CNFs. 6SC–CNF-PBAT showed a further 20% increase in stiffness compared to SC–CNF-PBAT thanks to both the higher amount of the high-*T*_g_ copolymer compared to PBAT, and further individualization of CNFs due to highly hindered CNF–CNF interaction. On the other hand, the observed loss of deformability (ε_b_ of 6SC–CNF-PBAT ≈ 75%, Table S5) can be ascribed to the larger and poor short copolymer–PBAT interface, further confirming the relevance of the entanglements.

Edlund et al.^23^ extruded 5 wt % CNFs with PBAT in the dry state and admicellar polymerization of fatty acrylate polymers was tested as a compatibilization strategy. Their nanocomposite with modified CNFs shows a 35% increase in PBAT Young’s modulus and a 38% reduction in deformation. Few other studies have reported the incorporation of modified CNFs in PBAT, with CNF contents below 2 wt %.^20,21^ At such low amounts, the embrittlement of PBAT is minimal and the stiffening and reinforcing effects are lower than those reported in our study as well as in Edlund’s work.^23^

All of the tensile-tested samples displayed stress-whitening in the elongated region attributed to crazing. As an example, Figure 4c shows the SEM of a fractured tensile specimen of LC–CNF-PBAT captured on a cryo-fractured surface along the tensile direction. Several elongated voids, surrounded by PBAT fibrils, are visible in the microscopy, nucleated especially around microscopical CNF agglomerates. Crazing can contribute to the toughening of the nanocomposites,^31^ and it corroborates the high deformations achieved even by CNF-PBAT at such relatively high nanocellulose contents.

**Figure 5:**
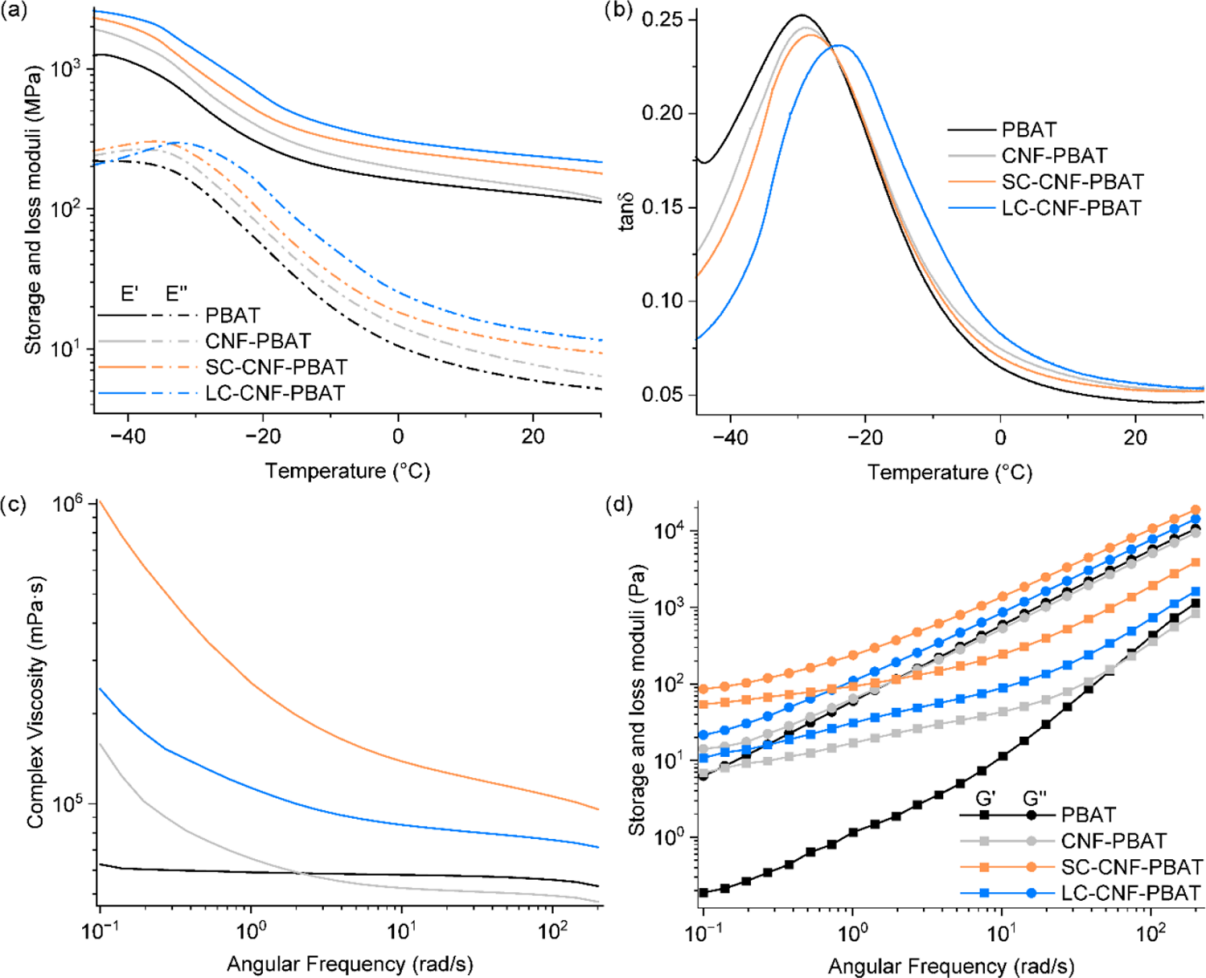
Representative curves of (a) storage and loss moduli in tension and (b) tan δ measured by DMTA in temperature sweeps at 1 Hz and 0.1% strain. Representative curves of (c) complex viscosity and (d) shear storage and loss moduli measured in frequency sweeps by rotational rheometry at 150 °C and 1% strain.

The dynamic mechanical properties were measured in temperature sweeps (from −45 to 30 °C) by DMTA in the tension mode. LC–CNF-PBAT has the largest storage modulus both in the glassy and rubbery regions (Figure 5a), in agreement with the stiffness measured by tensile testing. This nanocomposite also shows the lowest loss factor (tan δ) and the largest shift in the glass transition temperature of PBAT (from −36 °C for neat PBAT to −31 °C for LC–CNF-PBAT) (Figure 5b). A reduction in the loss factor magnitude is an indication of lower energy dissipation while the increase of glass transition is connected to restricted chain mobility,^32,33^ both consequences of improved dispersion and interfacial adhesion between the CNFs and the matrix in the presence of the long copolymer.

Rotational rheometry in the melt state (at 150 °C) provided further information about the molecular interactions of the nanocomposites. In the range of frequencies tested, PBAT shows a plateau of complex viscosity values, indicating typical Newtonian behavior (Figure 5c). In the nanocomposites, the plateau is replaced by a shear-thinning region at low frequencies due to progressive CNFs orientation, with an increase in complex viscosity below 2 rad/s. The nanocomposites with the copolymers display a similar trend but with an upward shift in viscosity values at all frequencies, highest for SC–CNF-PBAT. This result supports the hypothesis of a larger number of particles in SC–CNF-PBAT (Figure 2), in which a part of them is still individualized in the melt leading to larger interfaces compared to the ones present in LC–CNF-PBAT and CNF-PBAT. While some of the nanoparticles are expected to be adsorbed onto CNFs, a fraction is conceivably hypothesized to be free in the nanocomposite creating more interfaces with the matrix. The interaction between PBAT, CNFs, and the short copolymer, with significantly higher *T*_g_ than the matrix (123 vs −33 °C), can restrict the PBAT chains’ mobility in the melt, resulting in increased viscosity and moduli in the entire frequency range. In this case, the chain entanglement is not supposed to play a role in the rheological properties as the short hydrophobic tail was designed below the entanglement point with PBAT. Instead, LC–CNF-PBAT shows a lower decrease in complex viscosity before reaching the plateau, pointing at the effect of the entanglement of the hydrophobic long tails that counteracts the CNFs orientation and results in a broader viscosity plateau from around 3 rad/s.

The recorded storage and loss moduli indicate a predominant viscous character of PBAT (*G*″ > *G*′), evident also for the nanocomposites but with a reduced gap between *G*′ and *G*″, demonstrating relatively higher elasticity (Figure 5d). Overall, the presence of CNFs imparts rigidity, increasing the moduli and viscosity, and a quasi-plateau of the moduli at lower frequencies indicates the formation of an interconnected structure.^34^ While at lower frequencies the nanocomposites moduli vary with a smaller slope than PBAT, at higher frequencies (>20 rad/s) the moduli have a slope similar to the neat matrix. At higher frequencies, the interactions between the matrix and the CNFs are diminished due to orientation. CNF-PBAT approaches the viscoelastic behavior of neat PBAT, confirming poor dispersion and interface adhesion.

Overall, both copolymers increase the rheological properties of the nanocomposites, which can be ascribed to enhanced interfacial interactions. These can be due to the presence of stiffer nanoparticles (copolymers and CNFs), improved adhesion between PBAT and CNF, and enhanced dispersion of CNFs thanks to the adhesion to the hydrophilic anchor, designed to be identical for both copolymers. Rotational rheology helps to discern among these mechanisms, indicating a different behavior of the nanocomposites. The short copolymer led to an order of magnitude increments of viscosity and moduli at low frequencies and higher shear-thinning. This behavior has been ascribed to a higher number of nanoparticles, overcoming the lack of entanglement with PBAT. For the long copolymer, in addition to the higher CNF dispersion due to the effect of the hydrophilic anchor, the increase in viscosity and moduli and the reduced shear-thinning are attributed to the chain entanglements between the hydrophobic block and the PBAT. Despite the efforts to discern the effect of entanglements, it was not possible to control the number of nanoparticles formed by self-assembling of the two copolymers, which results in an additional interaction in the case of the short copolymer.

**Figure 6:**
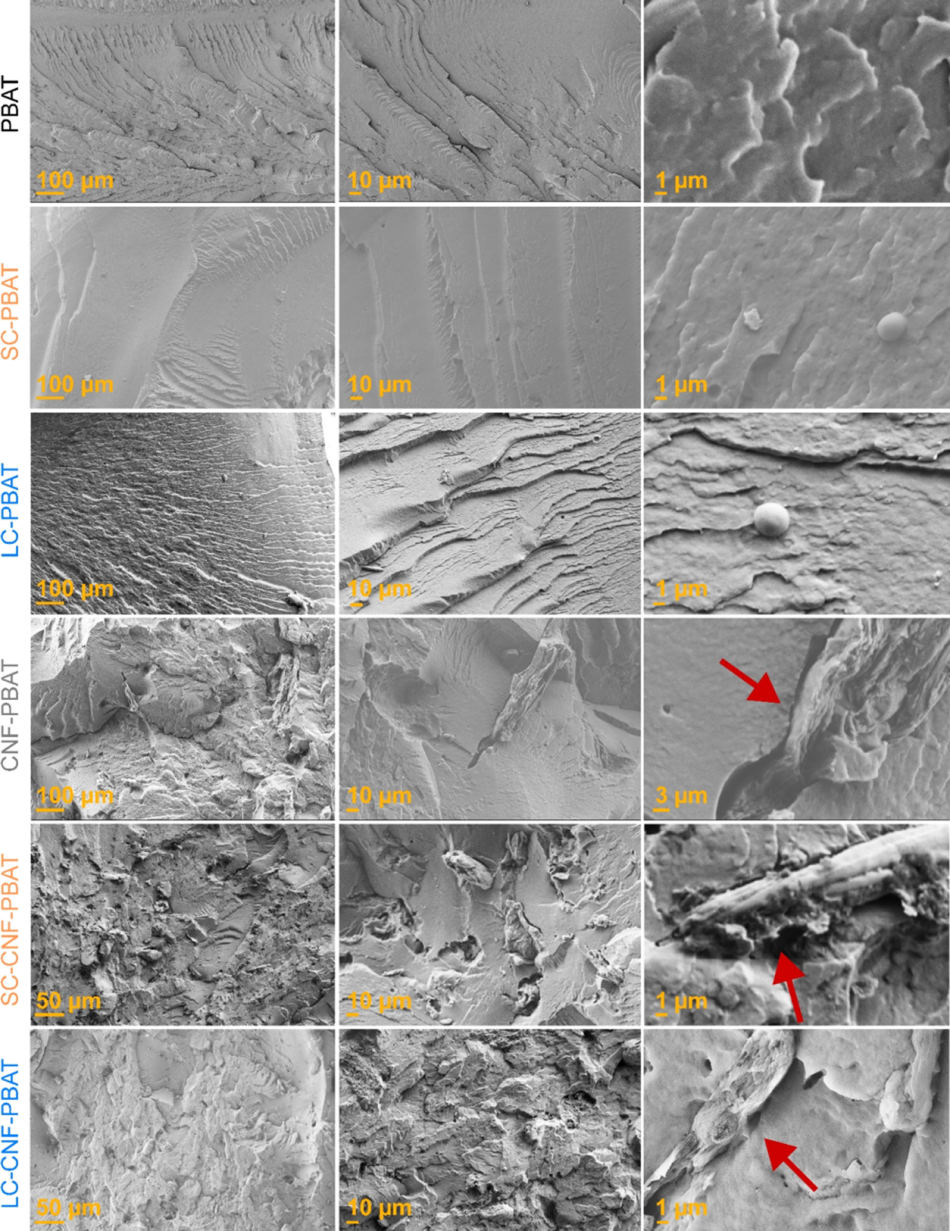
SEM micrographs of cryo-fractured nanocomposites and references. The largest magnification captured for CNF-PBAT is lower than that for the other materials for better visualizing the large agglomerates of the nanocomposite. The red arrows point to the identified CNFs.

The cryo-fractured surfaces of the nanocomposites and their references were characterized via SEM (Figure 6) to observe their morphology and relate it to the mechanical behavior. The surface of neat PBAT is smooth and featureless. The surfaces of SC-PBAT and LC-PBAT are equally smooth, but they show the presence of spherical particles with consistent dimensions, which indicate a phase separation of copolymer aggregates from PBAT. When the copolymers are adsorbed on CNFs, the spherical particles cannot be observed, indicating an interaction with CNFs that limits the copolymers’ aggregation and phase separation. As shown in Figure S6, the copolymers soften at the processing temperature above their glass transition. The ionic bonding between the charged PDMAEMA and CNF surfaces is hypothesized to drive the unfolding of the micelles on the CNF and there is no evidence of CNF-copolymers debonding after melt processing.^14^ Conversely, in the case of the references produced only with PBAT and the copolymers, there is no ionic bonding formed, as no CNF is present, so the copolymers phase-separate from the matrix in the melt. The surface of CNF-PBAT shows large flat regions with microscopic agglomerates of CNFs surrounded by voids as a consequence of the poor dispersion and poor interaction with the matrix, also confirmed by the reduction in PBAT deformation. The nanocomposites with the two copolymers present smaller CNF agglomerates than those of CNF-PBAT, confirming a better CNF dispersion. No debonding is observed in LC–CNF-PBAT, indicating that the long copolymer improves not only the dispersion but also the CNFs’ adhesion to the matrix, thus preserving PBAT ductility.

## Conclusions

Two waterborne anchor–tail nanomicellar diblock copolymers were successfully synthesized and applied for the interface engineering of cellulose nanofibril nanocomposites. The interface between CNFs and PBAT is replaced by two new interfaces, CNF–copolymer and copolymer–PBAT. The cationic anchor block was synthesized by the polymerization of DMAEMA and its length was maintained constant to generate dynamic ionic bonding with negatively charged oxidized CNFs. To mediate the copolymer–PBAT interface, two different tail lengths were molecularly engineered for the hydrophobic block to evaluate the effect of chain entanglement.

All of the results of the nanocomposites prepared with engineered copolymers achieved better performance than the ones prepared merely with unmodified CNFs. The adsorption of the nanomicelles onto the CNF surface is demonstrated by scanning and transmission electron microscopies. This designed CNF–copolymer interaction led to improved CNF dispersion in the nanocomposites, as validated by the morphological analysis. Consistently, stiffening and preservation of a high level of deformability of the nanocomposites prepared with the short and the long diblock copolymers were registered in the tensile tests, demonstrating the effectiveness of the dynamic ionic bonding. The nanocomposites with both copolymers displayed increased storage and loss moduli in the glassy and rubbery regions of PBAT, measured by a dynamic mechanical thermal analysis. The analysis also revealed that the long copolymer led to a shift of the glass transition to higher temperatures and a reduction in the loss factor, both indicating an improved interface between the CNF and the matrix. The morphological analysis of the nanocomposites indicates a better adhesion for the long-tail copolymer to PBAT, which explains the improved thermomechanical properties of LC–CNF-PBAT compared with the other nanocomposites. These results point out that a larger chain length favors the entanglement with the matrix and suggests the use of long hydrophobic tails for the mediation of the CNF-matrix interface. However, the presence of few pull-outs and debonding also in the compatibilized nanocomposite underlines the lack of miscibility between the hydrophobic tail of the copolymers and PBAT and suggests the screening of other macromolecular architectures that are potentially miscible with the matrix. This study provides insights into the effectiveness of a waterborne third component at the CNF–matrix interface and its structure–property relationship can serve as a benchmark for the molecular engineering of micellar block copolymers.

## Methods

### Materials

Deionized water was used unless stated otherwise. Ultrapure water (Milli-Q) with a resistivity of 18.2 MΩ·cm at 25 °C was obtained from a Millipore Milli-Q purification system.

2-(Dimethylamino)ethyl methacrylate (DMAEMA, 98%) and methyl methacrylate (MMA, 99%) were purchased from Merck and purified from the inhibitor by passing them through a column of activated basic aluminum oxide. Aluminum oxide (90 active basic), chloroform (CDCl3, ≥99%), copper(I) chloride (CuCl, ≥99%), 1,1,4,7,10,10-hexamethyltriethylenetetramine (HMTETA, 97%), methyl iodide (≥99.0%, MeI), and 2,2,6,6-tetramethylpiperidine-1-oxyl (98%, TEMPO) were purchased from Merck and used as received. Acetone (98%), deuterated acetone (acetone-*d*_6_, 99.8%), deuterated chloroform (CDCl_3_, 99.8%), *N*,*N*-dimethylformamide (DMF, >99.9%), ethanol (EtOH, 96%), methanol (MeOH, ≥ 99.8%), and tetrahydrofuran (THF, ≥99%) were purchased from VWR Chemicals and used as received.

Cellulose nanofibrils (CNFs) were prepared by TEMPO-mediated oxidation^35^ of never-dried softwood dissolving pulp fibers, kindly donated by Domsjö Fabriker (Örnsköldsvik, Sweden). CNF dispersions at a concentration of 0.2 wt % in Milli-Q water were prepared by dilution and ultrasonication followed by centrifugation. The complete and detailed procedure was previously reported by Kaldéus et al.^36^ The obtained CNFs had a surface charge density of 500 μmol/g assessed by polyelectrolyte titration (PET). The average dimensions of CNFs extracted from softwood sulfite dissolving pulp were estimated by Fall et al.^37^ to be as follows: width of 4 nm and length in the range of 300–1000 nm.

Poly(butylene adipate-*co*-terephthalate) (PBAT) was purchased from Jinhui ZhaoLong High Technology Co. Ltd. (China), with a declared density of 1.26 g/cm^3^ and a melt flow index ≤5 g/10 min (ISO 1133) at 190 °C and 2.16 kg.

### Preparation of Copolymers

Amphiphilic diblock copolymers of DMAEMA and MMA were synthesized by atom transfer radical polymerization (ATRP) in two steps, followed by quaternization of the DMAEMA units to produce cationic polyelectrolytes: SC, i.e., short copolymer (*q*PDMAEMA_34_-*b*-PMMA_97_), and LC, i.e., long copolymer (*q*PDMAEMA_34_-*b*-PMMA_553_).

A PDMAEMA macroinitiator was synthesized and subsequently chain-extended with MMA in acetone with the following molar ratios for the monomer, initiator, catalyst, and ligand: 200/1/1/2 for SC and 400/1/1/2 for LC, according to a previously described procedure.^38^ The amounts used for the synthesis of SC are reported here as a representative example. PDMAEMA (13.7 g, 2.50 mmol), MMA (50.0 g, 499 mmol), and DMF as internal standard (9.13 g, 125 mmol) were added into a round-bottom flask placed in an ice bath under magnetic stirring. A solution of CuCl (247 mg, 2.50 mmol) and HMTETA (1.15 g, 4.99 mmol) in acetone (25.0 g) was added. After the flask was sealed with a rubber septum, argon was purged in the solution for 15 min. The reaction was allowed to start by placing the flask in an oil bath preheated to 50 °C. At 16% conversion of SC (*t* = 105 min) and 55% conversion of LC (*t* = 280 min), the reaction was stopped by placing the flask in an ice bath and exposing the mixture to air. The copper complexes were removed by passing the mixture through a column with basic aluminum oxide. The copolymers, dissolved in THF, were precipitated three times in 100 mL/g_polymer_ ice-cold methanol, dried under vacuum at room temperature, and finally stored at 4 °C in the dark.

Thereafter, the tertiary amines in the DMAEMA units of the diblock copolymers were quaternized by MeI. The copolymers (1.00 g, 2.25 mmol_DMAEMA_ for SC, and 1.00 g, 0.56 mmol_DMAEMA_ for LC) were solubilized in 1 wt % THF in a round-bottom flask, under magnetic stirring. 5 wt % MeI in THF (3 equiv per DMAEMA repeating unit) was added. The reaction proceeded at room temperature overnight. After that, THF and excess MeI were removed by rotoevaporation. SC and LC were dried under vacuum and stored at 4 °C.

To achieve self-assembly of SC and LC into spherical nanoparticles in water, the copolymers were dissolved in 1 wt % THF and the solution was added dropwise to Milli-Q water under magnetic stirring. Water dispersions of SC and LC, with a concentration of approximately 0.1 wt %, were obtained after dialysis for 3 days against Milli-Q water, using Fisher Scientific Spectra/Por dialysis tubing (6–8 kDa molecular weight cutoff).

### Adsorption of Copolymers onto CNFs

For the homogeneous adsorption of the copolymers onto CNFs, dilute water dispersions of SC and LC (approximately 0.1 wt %) were slowly added dropwise to a 0.15 wt % CNF dispersion, under magnetic stirring at room temperature. CNFs and copolymers were mixed in the weight ratios reported in Table 2. The dispersions of CNFs and copolymers were directly used for further experiments without any washing.

### Extrusion of Nanocomposites

PBAT in the powder form was added to the dispersions of CNFs (0.15 wt %), SC (0.1 wt %), LC (0.1 wt %), or CNFs/copolymer mixtures according to the compositions reported in Table 2. Excess water was evaporated by heating the mixtures at 50 °C under magnetic stirring until the water content reached 7.5 g (50 wt % of the total solid fraction). The mixtures were extruded in an Xplore micro compounder MC15HT at 160 °C, at 30 rpm during a 5 min feeding, and at 100 rpm during a 5 min processing. PBAT powder was also extruded dry and with water as comparative references. The extrudates were injection-molded with an Xplore IM12 into dumbbell-shaped specimens, rectangular bars, and disks with a barrel temperature of 160 °C (mold at 25 °C), following an injection program of 5 s at 280 bar and holding 30 s at 420 bar.

### Characterization Techniques

#### Nuclear Magnetic Resonance Spectroscopy (NMR)

Structural characterization of the copolymers through ^1^H NMR was conducted using a Bruker Avance spectrometer (400 MHz), at room temperature, with acetone-*d*_6_ as the solvent. The spectra were acquired with 32 scans and a 1 s relaxation delay, and the signal of acetone-*d*_6_ at 2.05 ppm was used as a reference.

#### Size-Exclusion Chromatography (SEC)

For assessing molecular weight and polydispersity of the copolymers, SEC was performed in DMF with a Tosoh EcoSEC HLC-8320GPC equipped with an EcoSEC RI detector and three PSS PFG microcolumns (MicroGuard, 100 and 300 Å). The analysis was conducted at 50 °C with DMF as the eluent (0.2 mL/min) and toluene as the internal standard. The calibration was made using PSS poly(methyl methacrylate) standards. The chromatograms were normalized to their height.

The molecular weight and polydispersity of PBAT after extrusion with or without water were determined by SEC in CHCl_3_, in which both samples were fully soluble, on a Malvern Viscotek GPCmax instrument equipped with a Viscotek VE3580 RI detector and three Malvern columns (TGuard column, followed by two LT4000L linear mixed bed columns). The analysis was conducted at 35 °C with CHCl_3_ as the eluent (0.5 mL/min) and toluene as the internal standard. The calibration was made using TDS polystyrene standards. Before analysis, all sample solutions were filtered through Thermo Fisher Scientific Fisherbrand PTFE membrane filters with 0.45 μm pore size.

#### Differential Scanning Calorimetry (DSC)

The thermal transitions and crystallinity were assessed by DSC with a Mettler Toledo DSC 2 calorimeter equipped with an HSS7 sensor and a TC-125MT intercooler. The copolymers were analyzed following a heating/cooling/heating temperature profile from −80 to 180 °C, at a heating rate of 10 °C/min, under N_2_ at a 50 mL/min flow rate. A similar method was used for the extruded materials reaching 200 °C instead of 180 °C. The degree of crystallinity (χ) was calculated according to eq 1
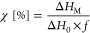
1where Δ*H*_M_ is the specific melting enthalpy, Δ*H*_0_ is the melting enthalpy of 100% crystalline PBAT (114 J/g^39^) and f is the weight fraction of PBAT.

#### Thermogravimetric Analysis (TGA)

The CNFs and copolymers were analyzed by TGA with a Mettler Toledo TGA/DSC 1 thermogravimetric analyzer. The samples were analyzed under a nitrogen atmosphere (with 50 mL/min flow) at a heating rate of 10 °C/min from 40 to 700 °C. An additional isothermal step of 5 min at 100 °C was added to remove moisture. The CNFs were further characterized with an isothermal program at 160 °C for 20 min under O_2_ to simulate their thermal degradation during melt processing. The thermal stability of the extruded samples was studied by TGA with a TGA/DSC 3+ Star system (Mettler Toledo). Approximately 5 mg samples were tested in alumina crucibles, with a first isotherm at 70 °C for 15 min to evaporate residual moisture. Then the samples were heated to 500 °C at a heating rate of 5 °C/min under N_2_ at a 50 mL/min flow rate. The temperature of thermal degradation onset (*T*_5%_) was measured as the temperature corresponding to the onset of 5% weight loss after moisture removal. The degradation temperature (*T*_d_) was defined as the peak temperature in the derivative thermogravimetric curve.

#### Scanning Electron Microscopy (SEM)

Field emission SEM (FE-SEM) micrographs of CNFs and copolymers were acquired using a Hitachi S-4800 microscope, with an accelerating voltage set to 1 kV. Specimens were prepared by dipping Topsil silicon wafers (cleaned by consecutive rinsing with Milli-Q water and EtOH, dried with nitrogen, and coated with poly(vinyl amine)) in water dispersions of the analyte. The samples were sputter-coated at a current of 80 μA with Pt/Pd for 10 s using a Cressington 208RH sputter coater. FE-SEM micrographs of the copolymers at 30× magnification were processed using the ImageJ software (NIH) to calculate the size distribution of the micelles. Nanoparticles with diameters below 5 nm were not included in the calculations to exclude background noise.

The extruded materials were analyzed with a field emission gun SEM (FEG-SEM). The injection-molded samples and a LC–CNF-PBAT tensile-tested sample were cryo-fractured in liquid nitrogen. The surfaces were gold-sputtered for 60 s at 10 mA. The fractured surfaces were investigated with a Zeiss Sigma Ultra 55 FEG-SEM instrument with 10 kV accelerating voltage.

#### Total Nitrogen Analysis

A PAC Antek MultiTek elemental analyzer was used to conduct total nitrogen analysis of the copolymers. A calibration curve (Figure S15) was created by injecting 1–15 μL samples of the PDMAEMA macroinitiator dissolved in acetone (10 g/L). Six measurements were averaged to obtain each data point of the calibration curve. Thereafter, the copolymers were dissolved in acetone and analyzed. Based on the calibration curve, the mol_DMAEMA_/g_copolymer_ values were determined, from which the molar ratio between the two blocks was calculated and consequently the DP of PMMA in the copolymers.

#### Polyelectrolyte Titration (PET)

The surface charge of CNFs and copolymers was assessed by the polyelectrolyte titration of dilute dispersions using a Particle Matrix Stabino unit. CNF dispersions were titrated with a solution of polydiallyldimethylammonium chloride. The copolymers were titrated with a solution of potassium poly(vinyl sulfate).

#### Tensile Testing

The injection-molded samples were conditioned for 48 h at 23 °C and 53% relative humidity before testing. A tensile speed of 2.5 mm/min (10%/min) was used to test at least 3 specimens for each material using a Zwick/Z2.5 tensile instrument (ZwickRoell) equipped with a load cell of 2 kN.

#### Optical Microscopy

The dispersions of CNFs and LC/CNF 1:1, SC/CNF 5:1, and SC/CNF 1:1 by weight were observed between glass slides with a ZEISS Axioscope A1 optical microscope in transmitted light mode.

#### Transmission Electron Microscopy (TEM)

A Hitachi HT7700 TEM instrument was used to image the dispersions of nanoparticles and CNFs. Nanoparticle dispersions (0.05 wt %) and mixtures with 0.05 wt % CNF dispersions were cast and dried on grids (200 square mesh). The acceleration power was 100 kV.

#### Dynamic Light Scattering (DLS)

The hydrodynamic diameter (*D*_H_) and polydispersity index (PdI) of the copolymers were determined with a Malvern Zetasizer NanoZS instrument at ambient temperature. Each value used is the average of three consecutive measurements on the same sample. The standard chosen for the size correlation was polystyrene latex, set by default from the instrument.

#### Dynamic Mechanical Thermal Analysis (DMTA)

Rectangular bars (25 × 5 × 1 mm^3^) cut from injection-molded specimens were tested in tension-film mode with a DMA Q850 (TA Instruments) apparatus equipped with an air chiller system. The bars were conditioned for 48 h at 23 °C and 53% relative humidity prior to testing. Strain sweep measurements at room temperature and 1 Hz were carried out to determine the linear viscoelastic region of the materials, and 0.1% strain was selected within linearity. Temperature sweeps were performed at 1 Hz and 0.1% strain between −45 and 30 °C at a heating rate of 2 °C/min. Before the temperature ramp, the samples were soaked for 2 min at −45 °C.

#### Rotational Rheology

Dynamic rheological measurements were carried out on an Anton Paar MCR 702 rheometer with a parallel plate geometry (25 mm diameter). Injection-molded disks (25 mm diameter, 2 mm thickness) were conditioned for 48 h at 23 °C and 53% relative humidity prior to testing. The disks were maintained for 2 min at 150 °C and then tested isothermally at a gap of 1 mm. Frequency sweep tests were carried out in an angular frequency range from 0.1 to 100 rad/s at an applied strain of 1% within the linear region.

## Supporting Information Available

Molecular weight distributions of block copolymers (Figure S1); second heating of DSC of the copolymers (Figure S2); TEM micrographs of the copolymers (Figure S3); SEM micrographs of the copolymers (Figure S4); ImageJ analysis of the SEM micrographs (Figure S5); hydrodynamic diameter and polydispersity index of copolymer nanoparticles by DLS (Table S1); SEM micrographs of LC–CNF at room temperature and after annealing (Figure S6); TGA of CNFs and copolymers (Figure S7); onset of degradation temperature of CNFs and the copolymers (Table S2); TGA of CNFs with an isothermal program in O_2_ (Figure S8); molecular weights and polydispersity of PBAT extruded dry or with water (Table S3); SEC traces in chloroform of PBAT extruded dry or with water (Figure S9); photographs of injection-molded specimens of PBAT and the nanocomposites (Figure S10); DSC and TGA of the nanocomposites and their references (Figure S11); thermal properties of the extruded materials (Table S4); tensile properties at room temperature of the injection-molded samples (Table S5); photographs of vials containing CNF, LC/CNF 1:1, SC/CNF 5:1, and SC/CNF 1:1 by weight (Figure S12); optical microscopies of CNF, LC/CNF 1:1, SC/CNF 5:1, and SC/CNF 1:1 by weight (Figure S13); SEM of 6SC–CNF-PBAT (Figure S14); calibration curve of total nitrogen analysis on samples of PDMAEMA macroinitiator (Figure S15) (PDF)
