# Supplementary material for: Design of Extruded Nanostructured Composites via Decoupling of the Cellulose Nanofibril/Poly(butylene adipate-co-terephthalate) Interface
Source: ACS Appl Mater Interfaces. 2024 Dec 23;17(1):2602–14. doi: 10.1021/acsami.4c17899 (PMC11783528; doi:10.1021/acsami.4c17899)
Supplement: Supplementary file 1 — am4c17899_si_001.pdf [file am4c17899_si_001.pdf]

Supporting information

Design of extruded nanostructured composite via decoupling of the cellulose nanofibril/poly(butylene adipate-co-terephthalate) interface

Angelica Avella[a,b,°], Maria Rosella Telaretti Leggieri[c,°], Alexandros Efraim Alexakis[c,d], Eva Malmström[c,d] and Giada Lo Re[a,b,*]

[a] Department of Industrial and Materials Science, Chalmers University of Technology, SE-412 58 Gothenburg, Sweden.
[b] Wallenberg Wood Science Centre, Chalmers University of Technology, Kemigården 4, SE-412 96 Gothenburg, Sweden.
[c] Division of Coating Technology, Department of Fibre and Polymer Technology, School of Engineering Science in Chemistry, Biotechnology and Health, KTH Royal Institute of Technology, Teknikringen 56–58, SE-100 44 Stockholm, Sweden.
[d] Wallenberg Wood Science Centre, Department of Fibre and Polymer Technology, KTH Royal Institute of Technology, Teknikringen 56–58, SE-100 44 Stockholm, Sweden.
*Corresponding author: giadal@chalmers.se
[°]A.A. and M.R.T.L. have contributed equally to this work.

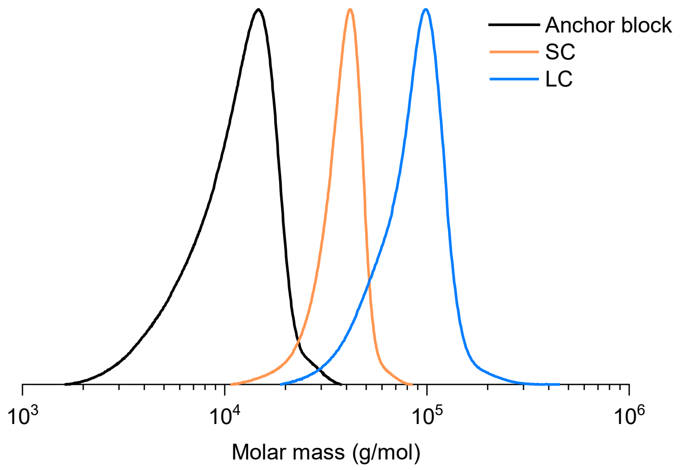

Figure S1. Molecular weight distributions of block copolymers, determined by SEC in DMF before quaternization: anchor block (PDMAEMA macroinitiator), SC ($PDMAEMA_{34}$-b-$PMMA_{97}$) and LC ($PDMAEMA_{34}$-b-$PMMA_{553}$).

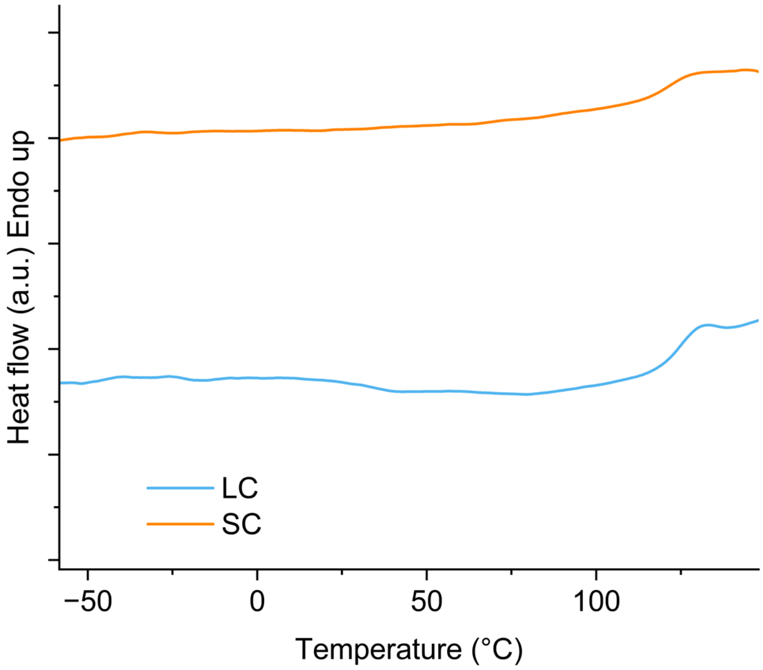

Figure S2. Second heating of DSC of the long and short copolymers indicating their glass transition temperatures.

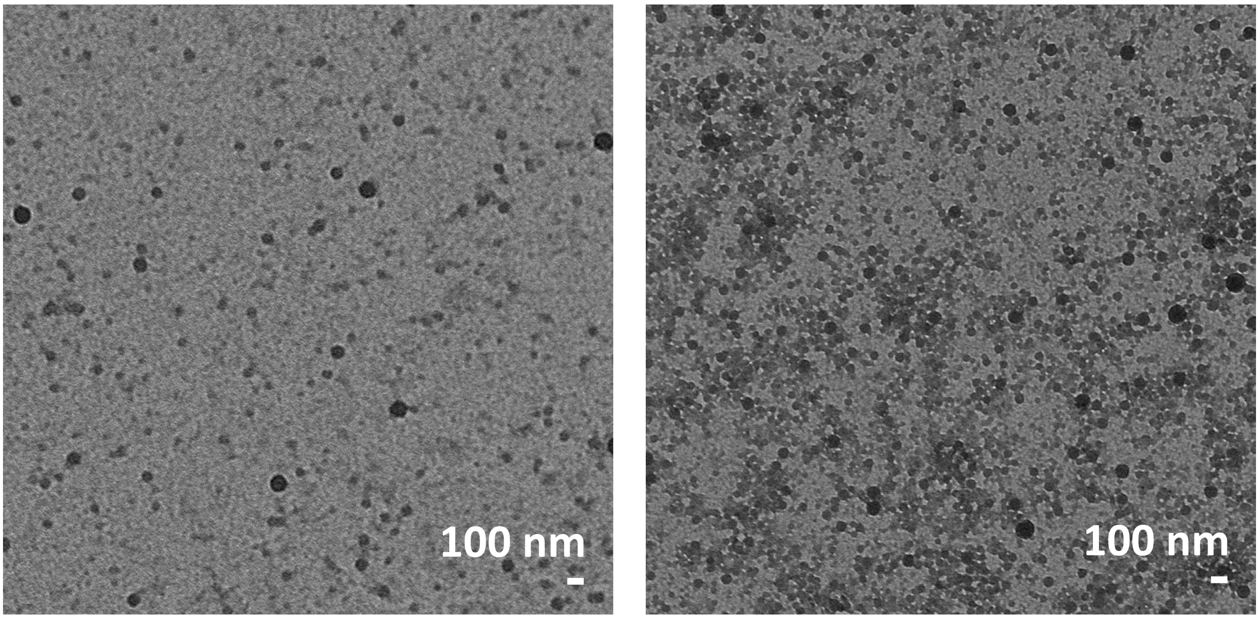

Figure S3. TEM micrographs of SC (left) and LC (right).

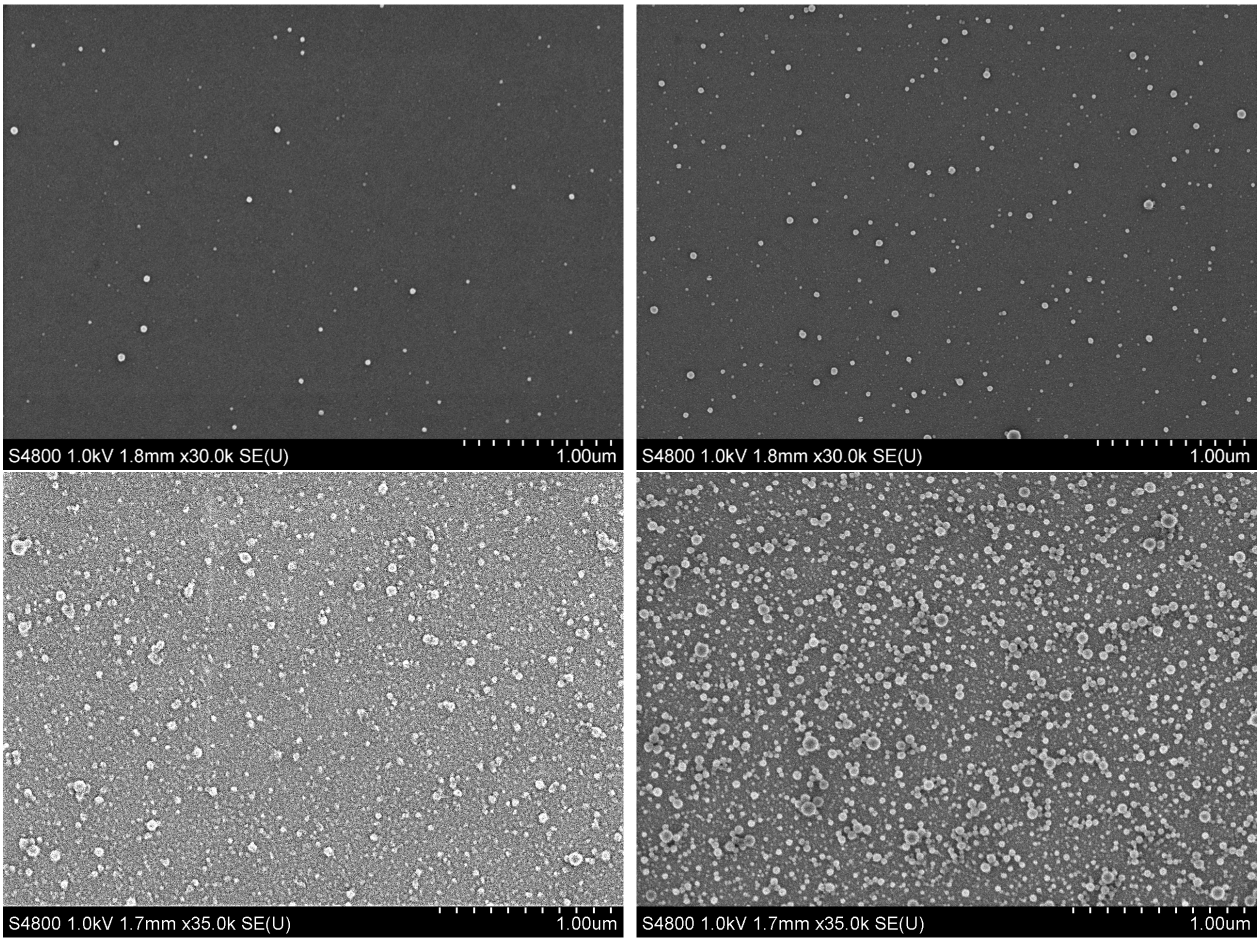

Figure S4. SEM micrographs of SC (left) and LC (right) from 0.05 wt.% water dispersions (top) and 0.1 wt.% water dispersions (bottom).

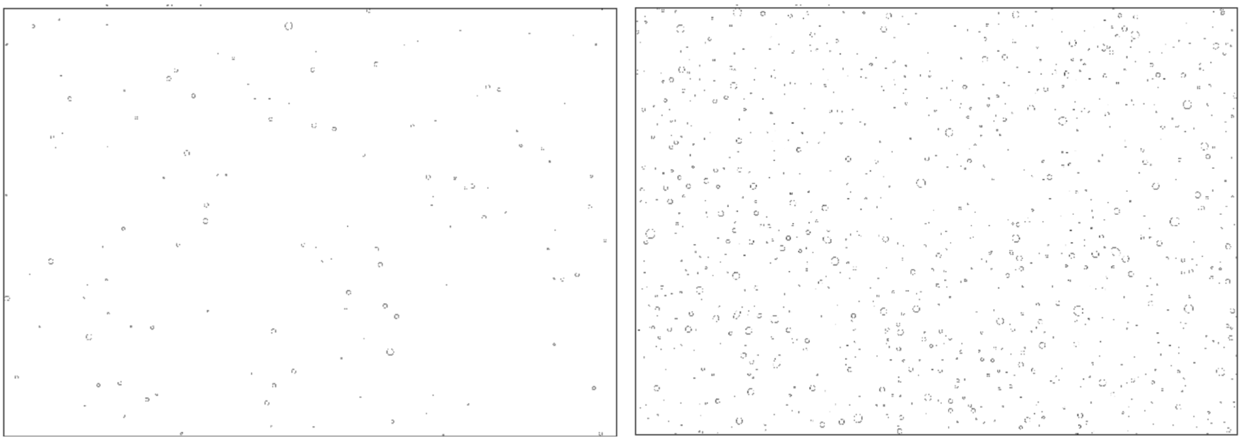

Figure S5. ImageJ results where nanoparticles in SEM micrographs (Figure S5) were approximated to ellipses to calculate their dimensions.

Table S1. Hydrodynamic diameter (D_H) and polydispersity index (PdI) of copolymer nanoparticles assessed by DLS in deionized water.

**Sample**	D_H **(nm)**	***PdI***
**SC**	101 ± 3	0.22 ± 0.01
**LC**	94 ± 3	0.18 ± 0.01

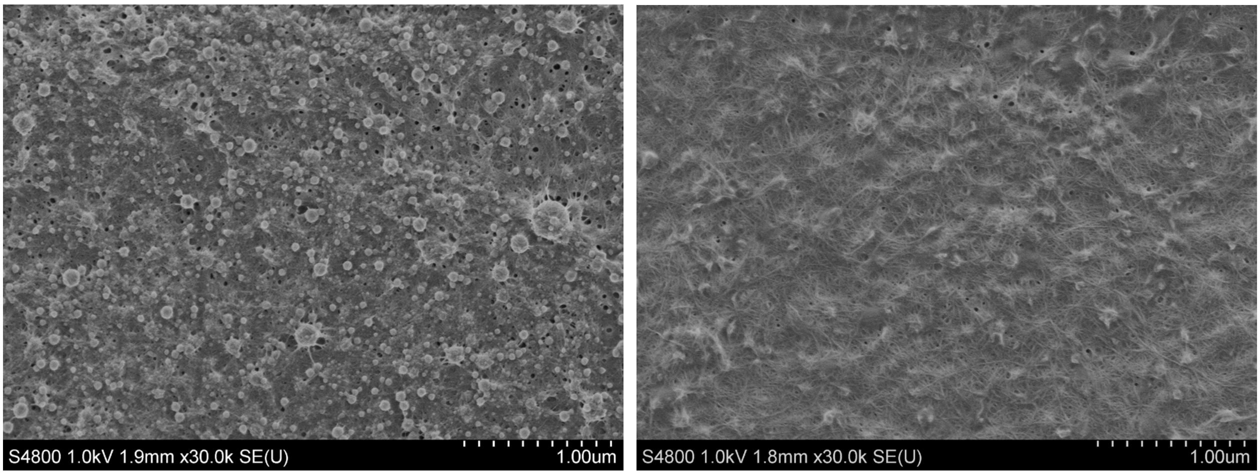

Figure S6. SEM micrographs of LC-CNF at room temperature (left) and after annealing at 160 °C for 20 min (right).

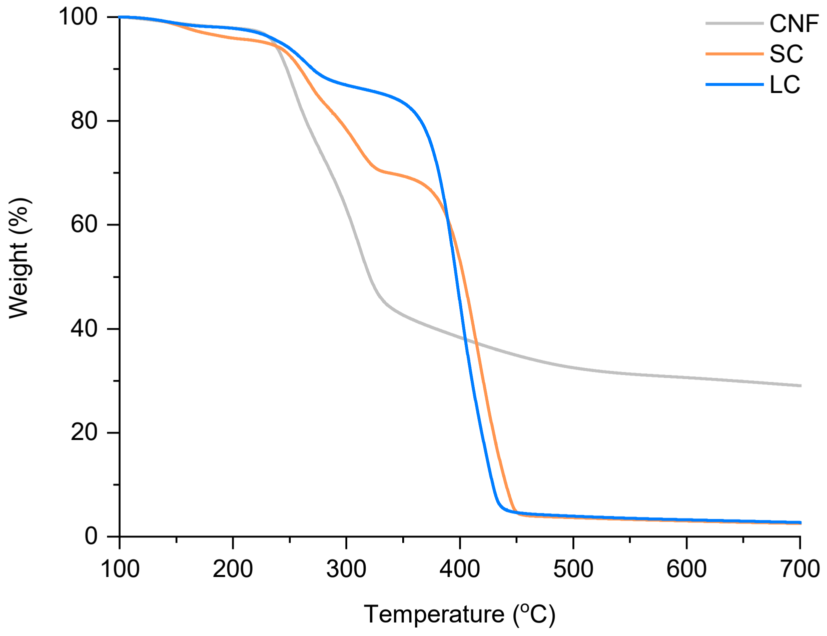

Figure S7. TGA thermograms under N_2 of CNFs and the copolymers.

Table S2. Onset of degradation temperature (at 5% weight loss) of CNFs and the copolymers.

**Sample**	$T_{5\%}$ **(°C)**
**CNF**	236
**SC**	228
**LC**	242

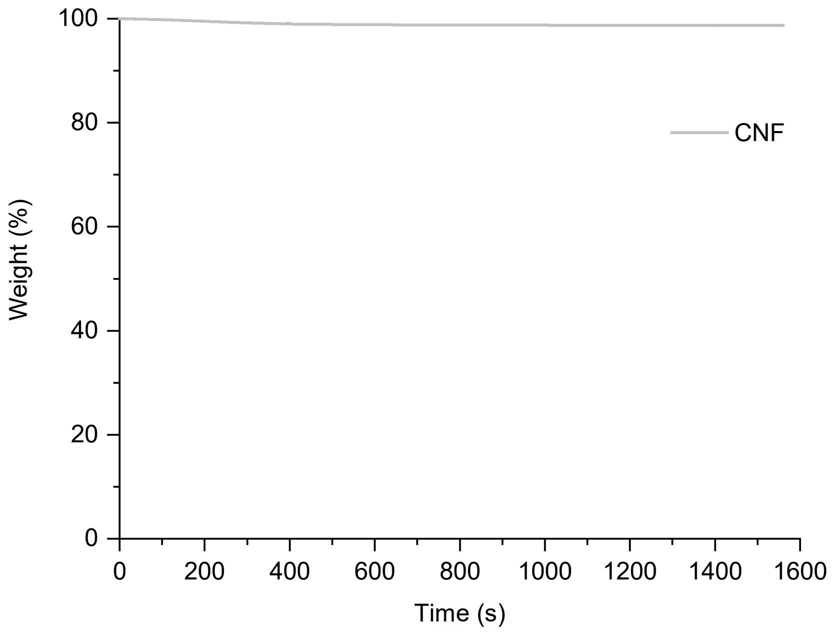

Figure S8. TGA of CNFs with isothermal program at 160 °C for 20 min in O_2.

Table S3. Molecular weights and polydispersity of PBAT extruded dry or with water (50 wt.% of PBAT), measured by size-exclusion chromatography in chloroform.

**Samples**	$\bar{M}_n$ **(Da)**	$\bar{M}_w$ **(Da)**	$Đ$
**PBAT$_{dry}$**	37 400	79 000	2.1
**PBAT**	31 100	71 800	2.3

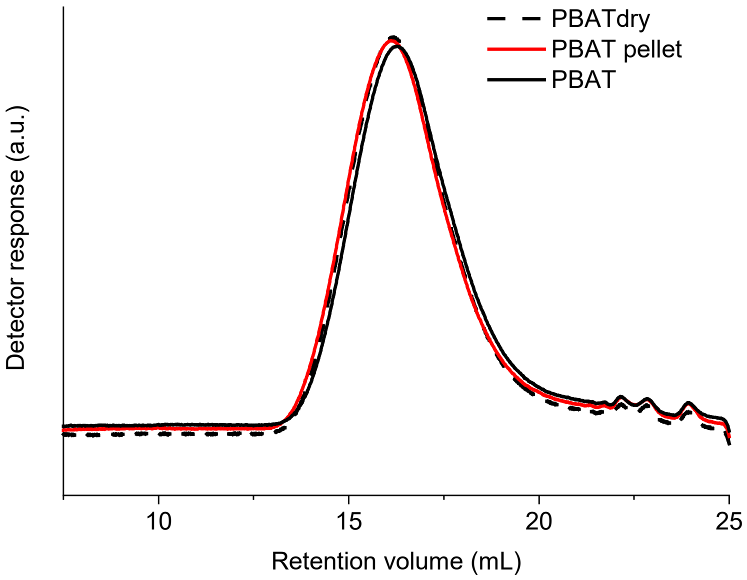

Figure S9. SEC traces in chloroform of PBAT powder extruded dry (PBATdry), PBAT pellet, and PBAT powder extruded in the presence of water (50 wt.% of the solid fraction).

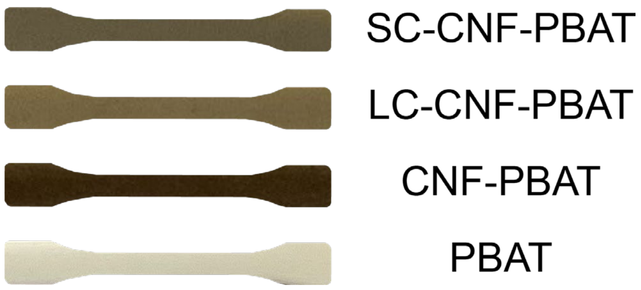

Figure S10. Photographs of injection-molded dumbbell-shaped specimens of PBAT and the nanocomposites.

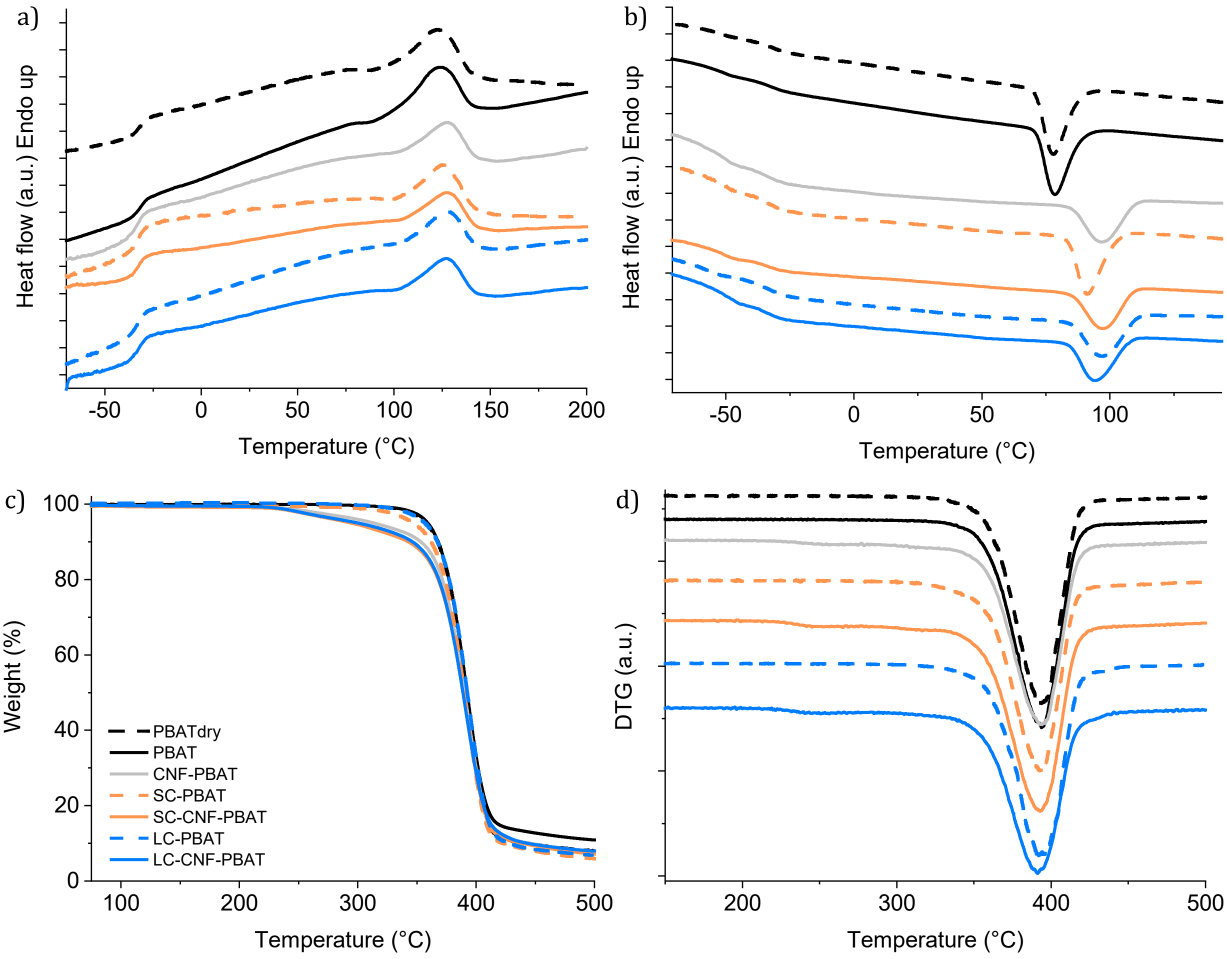

Figure S11. a) Second heating and b) cooling curves from DSC of the nanocomposites and their references. c) TGA and d) its first derivative.

Table S4. Thermal properties of *the extruded materials measured by DSC and TGA.*

**Samples**	T_g (°C)	T_m (°C)	ΔH_m (J/g)	χ_c (%)	T_c (°C)	$T_{5\%}$ (°C)	T_d (°C)	**Char (%)**
**PBAT$_{dry}$**	–33	123	13	12	78	358	395	8
**PBAT**	–31	124	13	12	78	359	393	11
**CNF-PBAT**	–33	127	9	8	97	313	394	8
**SC-PBAT**	–33	126	11	9	91	347	394	6
**SC-CNF-PBAT**	–33	128	9	8	97	295	393	7
**6SC-CNF-PBAT**	–33	128	10	9	95	310	392	8
**LC-PBAT**	–34	128	9	8	97	357	394	7
**LC-CNF-PBAT**	–32	127	8	8	94	300	391	8

Table S5. Tensile properties at room temperature of the injection-molded samples.

**Samples**	**Young's Modulus (MPa)**	**Ultimate tensile strength (MPa)**	**Elongation at break (%)**
PBATdry	52 ± 1	15 ± 1	809 ± 48
PBAT	56 ± 2	16 ± 0	1072 ± 71
CNF-PBAT	59 ± 10	9 ± 2	204 ± 110
SC-PBAT	50 ± 1	16 ± 1	1028 ± 59
SC-CNF-PBAT	80 ± 2	8 ± 0	209 ± 35
6SC-CNF-PBAT	97 ± 3	9 ± 0	76 ± 1
LC-PBAT	61 ± 5	12 ± 1	671 ± 115
LC-CNF-PBAT	86 ± 5	11 ± 0	447 ± 85
3LC-3CNF-PBAT	60 ± 2	14 ± 0.5	800 ± 25

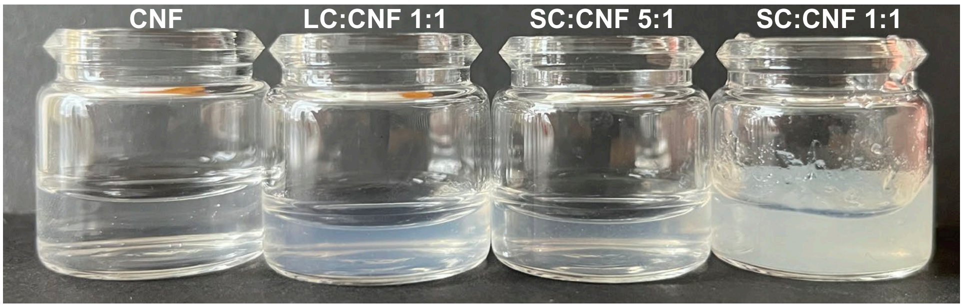

Figure S12. Photographs of vials containing CNF, LC:CNF 1:1, SC:CNF 5:1 and SC:CNF 1:1 by weight.

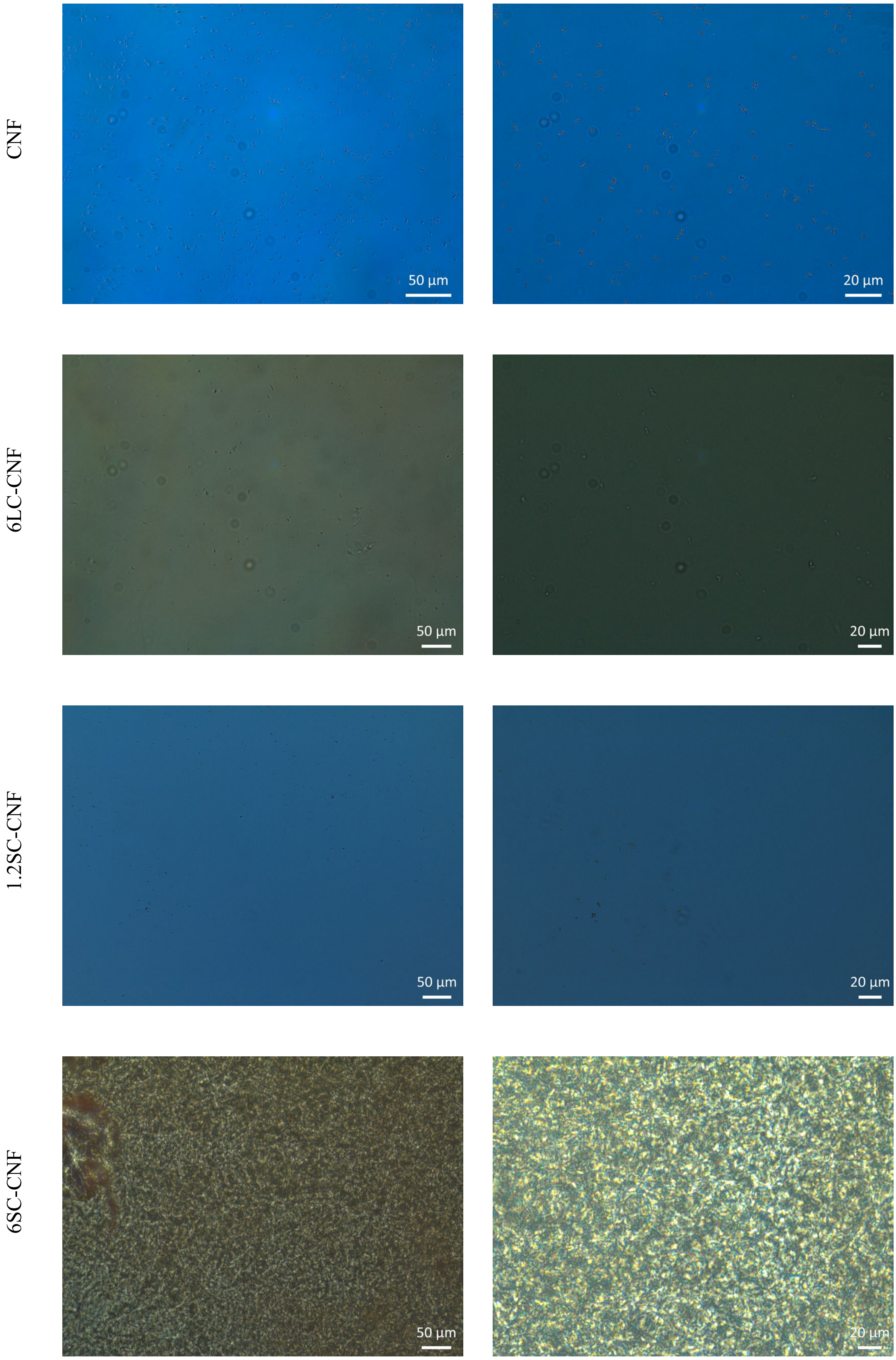

Figure S13. Optical microscopies at two magnifications of CNF, LC:CNF 1:1, SC:CNF 5:1 and SC:CNF 1:1 by weight.

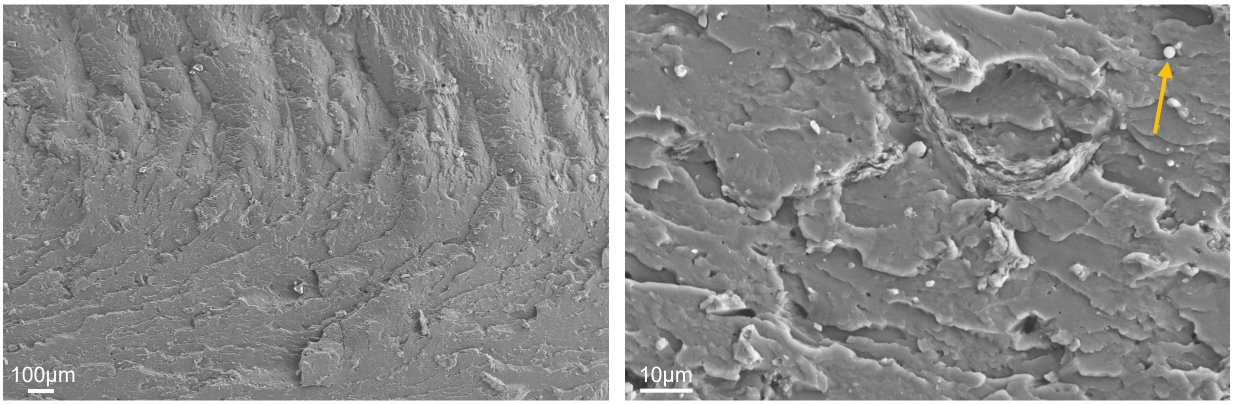

Figure S14. Scanning electron microscopies of cryo-fractured 6SC-CNF-PBAT at two magnifications.

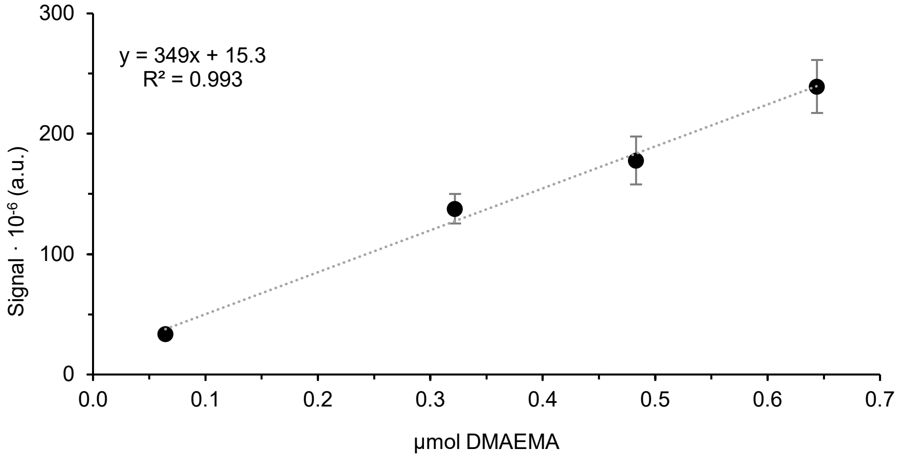

Figure S15. Calibration curve of total nitrogen analysis on samples of PDMAEMA macroinitiator.
